# Non-cerebral malaria: does such a thing exist?

**DOI:** 10.1590/0074-02760240223

**Published:** 2025-02-03

**Authors:** Beatriz Nogueira Siqueira-e-Silva, Luciana Pereira de Sousa, Pamela Rosa-Gonçalves, Rízia Maria da Silva, Yuri Chaves Martins, Patrícia Brasil, Cláudio Tadeu Daniel-Ribeiro

**Affiliations:** 1 Fundação Oswaldo Cruz-Fiocruz, Instituto Oswaldo Cruz, Laboratório de Pesquisa em Malária, Rio de Janeiro, RJ, Brasil; 2 Universidade Federal do Rio Grande do Norte, Centro de Biociências, Departamento de Microbiologia e Parasitologia, Natal, RN, Brasil; 3 Saint Louis University School of Medicine, Department of Anesthesiology, Saint Louis, MO, USA; 4 Fundação Oswaldo Cruz-Fiocruz, Instituto Nacional de infectologia Evandro Chagas, Laboratório de Doenças Febris Agudas, Rio de Janeiro, RJ, Brasil; 5 Ministério da Saúde, Secretaria de Vigilância em Saúde e Ambiente e Fundação Oswaldo Cruz-Fiocruz, Centro de Pesquisa, Diagnóstico e Treinamento em Malária, Rio de Janeiro, RJ, Brasil

**Keywords:** brain changes, cerebral malaria, cognitive and behavioral dysfunction, experimental malaria, non-cerebral malaria, systemic inflammation

## Abstract

Malaria, caused by *Plasmodium* spp., remains a major public health problem. Cerebral malaria is its deadliest form, with a 15-25% mortality rate, despite artemisinin-based treatments. In addition, the World Health Organization (WHO) strictly defines cerebral malaria as the presence of coma, 1 h after a seizure or the correction of hypoglycemia, in patients with *P. falciparum* parasitemia. Consequently, 25% of survivors experience neurocognitive and behavioral sequelae, particularly in children. However, more recently, neurocognitive and behavioral impairments were also reported in severe non-cerebral malaria, non-severe malaria, and even during asymptomatic *Plasmodium* infection. Such impairments have been observed in school-aged children, the elderly, and in animal models without classic cerebral malaria pathology. Additionally, mild vasogenic edema has been detected in neuroimaging of patients with severe non-cerebral and non-severe *P. falciparum* malaria. Therefore, given that approximately 98% of malaria cases in the world are non-severe, neurocognitive and behavioral sequelae may account for a significant proportion of global malaria morbidity. Taken together, these observations suggest that systemic inflammation from malaria, even without traditional cerebral malaria signs, can disrupt brain function and lead to long-term sequelae. We propose that the current definition of cerebral malaria may not fully capture the observed evidence and a new conceptualization is necessary to encompass these findings.

Despite affecting more than 260 million people and causing almost 600,000 deaths annually, malaria remains a neglected global public health problem primarily affecting poor countries.^1^ The burden of malaria extends beyond mortality. Malaria-related socioeconomic, neurocognitive, and behavioral morbidity in scholarly, professional, and personal daily life performance is often underestimated, particularly in non-severe cases.^2^ ^,^ ^3^ ^,^ ^4^ ^,^ ^5^

*Plasmodium falciparum*, the deadliest malaria parasite, is responsible for over 97% of global cases, of which 1 to 3% of cases may progress to severe forms, such as severe anemia, hypoglycemia, seizure, acute kidney injury (AKI) and cerebral malaria (CM).^1^ CM is characterized by coma, defined by using the “Blantyre coma score” (< 3) or “Glasgow coma score” (< 11), that persists for more than 1 h after termination of a seizure or correction of hypoglycemia, in a patient with *P. falciparum* parasitemia that has no other identified causes of encephalopathy.^6^ ^,^ ^7^ ^,^ ^8^

Approximately 25% of CM survivors, especially children, suffer from neurological (epilepsy, motor, visual, auditory, speech, and language difficulties), cognitive (memory and learning difficulties), and behavioral (inattention, hyperactivity, depression, impulsive and aggressive behavior) sequelae.^5^ ^,^ ^6^ ^,^ ^8^ ^-^ ^11^ Severe forms of malaria, aside from CM, can also cause long-term cognitive impairment (Figure).^5^ ^,^ ^12^ Additionally, AKI may increase the risk of neurocognitive and behavioral sequelae related to CM.^13^ ^,^ ^14^

**Figure:**
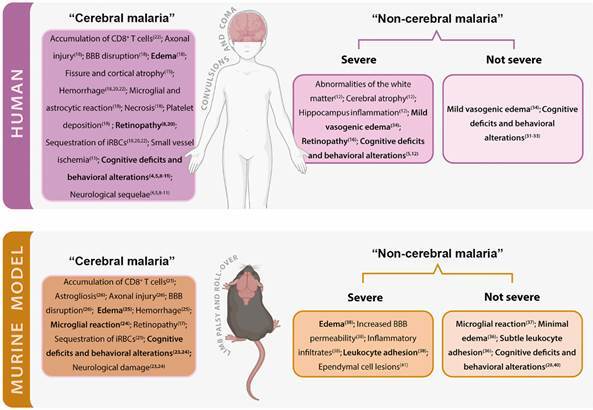
Brain changes recorded in “cerebral malaria” and in “non-cerebral malaria”. iRBCs: infected red blood cells; BBB: blood-brain barrier. Findings in bold indicate changes common to cerebral and non-cerebral malaria.

Magnetic resonance imaging (MRI) scans have revealed lesions indicative of small vessel ischemia and microinfarctions in periventricular areas of the brain of children who developed mental health disorders, including attention deficit and hyperactivity after CM.^15^ In children with persistent neurological sequelae (up to 21 months) after CM, MRI scans have also revealed cortical atrophy, fissures, and lesions in the gray matter and subcortical regions of the brain.^15^ In fundoscopy, alterations in the retina, a tissue of the central nervous system, can be observed in patients with CM and in other severe malaria syndromes.^16^ These changes were also observed in an experimental CM (ECM) model through retinal imaging and electroretinography.^17^

*Post mortem* human histopathological studies typically point to dense sequestration of infected red blood cells (RBC), multiple petechial hemorrhages and swelling mainly in the cortex and cerebellum.^18^ ^,^ ^19^ ^,^ ^20^ Reports of the presence of intense margination of mononucleated cells in the brain microvasculature are not universal in human CM, but T CD8^+^ cells have been observed in the brain of deceased children following CM.^21^ ^,^ ^22^

ECM models reproduce the neurocognitive damage observed in humans.^23^ Unlike human CM, histological studies in ECM show margination and adherence of activated inflammatory cells in the cerebral blood vessels, associated with perivascular edema and extravasation of RBC in the hippocampus, thalamus, midbrain, and cerebellum.^24^ ^,^ ^25^ In addition, demyelination, axonal injury,^26^ microglial reaction in the cortex and hippocampus,^24^ and increased inflammatory mediators in the brain^27^ were also shown in murine models of CM, which can explain alterations in cognitive function.

One may easily consider that a life-threatening clinical condition, with so many demonstrable histopathological and molecular changes in the brain, as well as long-lasting neurological and cognitive-behavioral sequelae, is characterized as a severe cerebral form of the disease. However, it may be less intuitive that non-severe malaria (nSM) can disturb the balance and harmony of brain function and be associated with long-term cognitive-behavioral sequelae.^3^ ^,^ ^4^ ^,^ ^26^ ^,^ ^28^

More recently, reports of cognitive impairment after nSM and even in asymptomatic infections have emerged.^29^ ^,^ ^30^ ^,^ ^31^ Since the vast majority (~ 98%) of malaria cases presents as nSM, cognitive impairment may account for a significant proportion of malaria-related morbidity worldwide. Children are the most affected, resulting in poor scholar and cognitive-behavioral performance.^30^ ^,^ ^31^ ^,^ ^32^ In addition, studies in aged individuals from a malaria endemic area in Brazil detected cognitive deficits up to eight months after infection.^33^ Furthermore, the presence of mild vasogenic edema in neuroimaging exams has been identified in patients with both severe non-cerebral and non-severe *P. falciparum* malaria.^34^

These sequelae of nSM are commonly linked to *P. falciparum* infection, but also do occur in *P. vivax* malaria.^31^ ^,^ ^32^ The deficits may be subtle and overlooked by some adult patients or affected child’s parents, unless they undergo cognitive-behavioral assessments. However, these deficits can be as detrimental to individual health and well-being as those reported in cerebral malaria patients.^30^ ^,^ ^31^

In experimental nSM, a very subtle adherence of monocytes, minimal focal cerebral edema^35^ ^,^ ^36^ and microglia reaction were observed in histopathology.^37^ *P. berghei* NK65-infected C57BL/6 mice, a model known not to cause ECM, shows mild brain changes as early as day 3 post-infection. These changes progress over time, with diffuse inflammatory infiltrate and intense hemorrhagic foci observed in histopathological evaluation at day 6 post-infection.^38^ However, Cimperman and colleagues^39^ did not observe brain pathology in hematoxylin and eosin-stained sections from mice on day 3 post-infection, in the same model.

Experimental models not only allow for the evaluation of cognitive-behavioral parameters in mildly infected animals, with no clinical sign of CM, but also allow the search for traditional histopathological alterations indicative of brain involvement.^36^ ^,^ ^40^ ^,^ ^41^ We adapted the classical model of ECM (C57BL/6 mice infected with *P. berghei* ANKA) to the study of nSM by treating animals at day 4 post-infection, prior to the development of any clinical sign of ECM.^40^ Using this adapted model, we observed long-term cognitive deficits and anxiety-like behavior as early as 12 and up to 150 days post-treatment.^28^ ^,^ ^40^ ^,^ ^42^ Previously, Guha and colleagues^37^ reported behavioral alterations (anxiety-like behavior and impaired social interaction) associated with experimental nSM caused by *P. chabaudi adami*. However, these alterations cannot be considered nSM sequelae, as the changes were recorded during (and not after) the acute infectious process, and thus in a host compromised by the systemic inflammation caused by the parasite. Therefore, as far as we know, our group was the first to report the presence of neurocognitive and behavioral sequelae in models of experimental nSM.^40^

More recently, the use of immunological and molecular methods suggests that the development of minimal brain changes during malaria infection could be sufficient to affect brain homeostasis and lead to the long-term neurocognitive and behavioral alterations registered after cure.^27^ ^,^ ^43^ ^,^ ^44^ We hypothesize that these minimal brain changes could be due to a deregulated systemic inflammatory response triggered by the parasite. However, the mechanisms behind how the marked systemic inflammation can cause the minimal structural changes present in nSM and generate the malaria-related neurocognitive and behavioral sequelae are still unknown.

Long-term cognitive sequelae are also observed after recovery from severe acute respiratory syndrome coronavirus 2 (SARS-CoV-2) infection^45^ that evolves with an intense inflammatory response, vascular dysfunction, and oxidative stress. Possible neurobiological events contributing to cognitive impairment in coronavirus disease 2019 (COVID-19) may involve neurovascular dysfunction, including blood brain-barrier (BBB) disruption, with consequent neuronal and glial dysregulation, neural circuit alteration^46^ and neurodegeneration, that are not necessarily present in nSM, in addition to the possible direct viral invasion.^47^

Neuroinflammation plays an important role in the pathophysiology of malaria. Although mild, dysregulation in microglial and astrocytes functions might influence neuroimmune crosstalk and signaling pathways promoting alterations in neuroplasticity, glutamatergic system and cognitive ability.^24^ ^,^ ^37^ ^,^ ^48^ These alterations may be mediated by pro-inflammatory cytokines, such as IL-1β (found at the prefrontal cortex at day 4 of infection in our experimental model of nSM - unpublished data) and that can be induced by hemozoin,^49^ by other cytokines that may be present at the brain even in low parasitemia during experimental infection^37^ ^,^ ^50^ and by extracellular vesicles from infected red blood cells influencing astrocyte function.^51^

Liver dysfunction, triggered by *Plasmodium* infection, may also play a role in the development of neurocognitive and behavioral sequelae, even in non-severe and asymptomatic cases.^52^ ^,^ ^53^ Such a dysfunction occurs during the erythrocytic stage of malaria infection, likely due to oxidative stress resulting from the release of substances like free heme, hemozoin, and other intracellular components into the bloodstream. Therefore, liver dysfunction caused by *Plasmodium* infection could lead to the accumulation of neurotoxins, including ammonia, due to decreased metabolism by the liver, that could cross the BBB and cause brain dysfunction like in cases of hepatic encephalopathy.

In conclusion, there is strong evidence that not only the severe CM syndrome, but all forms of malaria may have the potential to cause brain impairment. Therefore, it is time for the scientific and public health communities to recognize that the existing definition of cerebral malaria may not adequately account for the observed findings, requiring a revised conceptual framework. Although nSM is associated with low hospitalization rates, it may generate a substantial indirect economic burden in affected countries due to its impact on neurocognitive abilities, crucial for learning and work. Ultimately, reframing paradigms to recognize the fact that all cases of malaria have the potential to affect the brain could significantly increase the urgency of global malaria elimination efforts.
